# Evaluating the cytotoxicity and pathogenicity of multi-walled carbon nanotube through weighted gene co-expression network analysis: a nanotoxicogenomics study

**DOI:** 10.1186/s12863-022-01031-3

**Published:** 2022-02-17

**Authors:** Shameran Jamal Salih, Mohadeseh Zarei Ghobadi

**Affiliations:** 1 Department of Chemistry, Faculty of Science and Health, Koya University, KOY45, Koya, Kurdistan Region Iraq; 2 Institute of Biochemistry and Biophysics, University of Tehran, Tehran, Iran

**Keywords:** MWCNT, WGCNA, Carcinogenesis, Genotoxicity, DEGs

## Abstract

**Background:**

Multi-walled carbon nanotube (MWCNT) is one of the most momentous carbonaceous nanoparticles which is widely used for various applications such as electronics, vehicles, and therapeutics. However, their possible toxicity and adverse effects convert them into a major health threat for humans and animals.

**Results:**

In this study, we employed weighted gene co-expression network analysis (WGCNA) to identify the co-expressed gene groups and dysregulated pathways due to the MWCNT exposure. For this purpose, three weighted gene co-expression networks for the microarray gene expression profiles of the mouse after 1, 6, and 12-month post-exposure to MWCNT were constructed. The module-trait analysis specified the significant modules related to different doses (1, 10, 40, and 80 µg) of MWCNT. Afterward, common genes between co-regulated and differentially expressed genes were determined. The further pathway analysis highlighted the enrichment of genes including *Actb*, *Ube2b*, *Psme3*, *Ezh2*, *Alas2*, *S100a10*, *Ypel5*, *Rhoa*, *Rac1*, *Ube2l6*, *Prdx2*, *Ctsb*, *Bnip3l*, *Gp6*, *Myh9*, *Ube2k*, *Mbnl1*, *Kbtbd8*, *Riok3*, *Itgb1*, *Rap1a*, and *Atp5h* in immune-, inflammation-, and protein metabolism-related pathways.

**Conclusions:**

This study discloses the genotoxicity and cytotoxicity effects of various doses of MWCNT which also affect the metabolism system. The identified genes can serve as potential biomarkers and therapeutic candidates. However, further studies should be performed to validate them in human cells.

**Supplementary Information:**

The online version contains supplementary material available at 10.1186/s12863-022-01031-3.

## Background

Multi-walled carbon nanotubes (MWCNTs) are fiber-shaped carbon nanomaterials that have been employed in various commercial products like home appliances, electronics, vehicles, and also biomedical applications [1]. Besides several advantages of using MWCNTs in industrial applications, the most challenging issues are their contaminations, toxicity, and hazardous effects. Therefore, it is unavoidable to survey the toxicity characteristics of carbon nanotubes. The toxicity assessment of materials on the gene expression in the animal models can be generalized to the human target organ [2, 3].

Predominantly, workers who work in an industrial factory and are in contact with nanomaterials like MWCNTs, as well as consumers, are the main vulnerable groups to nanomaterial [4]. MWVNTs are light and simply aerosolized. Therefore, the workplace is a considerable source of human exposure to carbon nanotubes through inhalation or dermal contact. Moreover, the respiratory tract and damaged skin are the possible ways of exposure due to the small size and low density of MWCNTs. They may deposit in the mice’s lungs by inhalation or pharyngeal aspiration which causes histologic alterations including fibrosis and inflammation [5, 6].

Carbon nanotubes can induce asbestos-like pathogenicity with carcinogenic risk [7]. The main mechanism for the MWCNTs toxicity is the induction of oxidative stress through the production of free radicals such as reactive oxygen species (ROS) or reactive nitrogen species (RNS) [8–10]. Oxidative stress causes the generation or boosting of inflammation which is a remarkable risk factor for pulmonary carcinogenicity [9, 11]. Genotoxicity can also be generated by direct interaction of carbon nanotubes with genetic contents or by indirect harm from the induction of ROS [10]. MWCNTs induce the increase of profibrotic inflammatory mediators as well as pleural mesothelioma, lung carcinoma, and DNA damage responses [12–15]. The thin and entangled MWCNTs induce pulmonary inflammation due to lymphocytic aggregates, granulomas, and macrophage infiltration [16].

Weighted gene co-expression network analysis (WGCNA) is a practical and data reduction approach to find the co-regulated genes and correlation patterns across various specimens. The potential biomarkers and possible therapeutic targets may be detected through constructing co-expression networks and then identification of hub genes based on their biological functions and connection with other genes [17, 18]. WGCNA has several advantages including the transformation of gene expression data into a small number of co-expression groups (modules), finding the hub nodes in each module, and the associations between modules with the external traits. The co-regulated modules assist the annotation of results and relevant signaling networks that might be liable for phenotypic traits of interest [19]. The disadvantages of WGCNA are likely simplicity and producing false positives for cascades [20]. However, the co-expressed genes are often functionally related, governed by a similar transcriptional regulatory program, or involved in similar pathways. Therefore, they are of biological interest. This method has been employed to determine the co-regulated genes to find the possible pathogenesis mechanisms and potential biomarkers for various cancers, pathogens-caused diseases, and nanomaterials-caused toxicity [21–25]. In this study, we aimed to find the genotoxic co-expressed genes due to MWCNTs. For this purpose, we constructed the weighted gene co-expression networks for the gene expression profiling of different mouse groups exposed to various doses of MWCNT in different periods [26]. Further analyses led to the identification of co-regulated genes which implicate various pathways related to cytotoxicity and carcinogenicity.

## Results

### Construction of weighted gene co-expression network

**Fig. 1:**
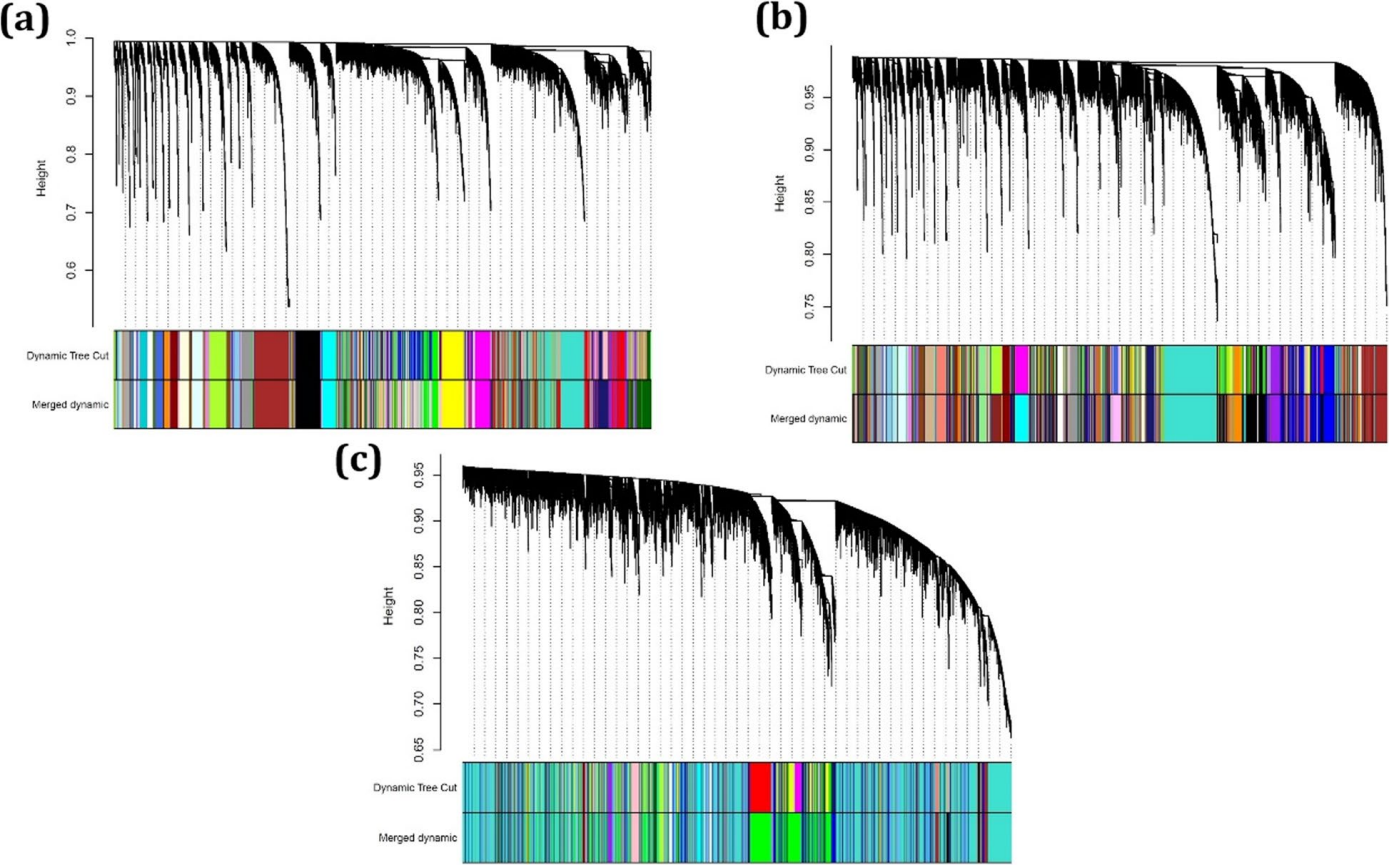
Dendrogram of genes clustered based on (1-TOM) with assigned module colors for **a)** MWCNT_1, **b)** MWCNT_6, and **c)** MWCNT_12. The color of rows indicates the module membership before and after merging modules

To build the co-expressed gene networks, the soft threshold powers β were determined as 4, 3, and 2 for MWCNT_1, MWCNT_6, and MWCNT_12, respectively. After calculating adjacency, TOM, dissTOM, hierarchical clustering, cutting branches and eventually merging close clusters, 39 modules for MWCNT_1, 48 modules for MWCNT_6, and 28 modules for MWCNT_12 were identified. Fig. 1a-c demonstrates the cluster dendrogram and modules before and after merging in which an inimitable color is ascribed to each module. In the dendrogram, the short vertical lines correspond to a gene. Moreover, branches of the dendrogram group show the densely interconnected and also highly co-expressed genes. The gene modules are related to the branches of the resulting dendrogram after merging the closed branches.

### Identification of specific modules in the MWCNT-exposed samples

To detect the specific non-preserved modules of three MWCNT-exposed groups, the Z_summary_ score and medianRank for each module were computed. The specific modules were those that have Z_summary_ < 2 and medianRank ≥ 8, in which turquoise, violet, steelblue, paleturquoise, darkgrey, orange, skyblue3, ivory, white, lightyellow, darkred, lightcyan, lightsteelblue1, mediumpurple3, royalblue, darkorange, darkorange2, orangered4, yellowgreen, greenyellow, grey60, darkolivegreen, darkturquoise were specific in MWCNT_1m; lightcyan, lightgreen, darkslateblue, paleturquoise, thistle2, sienna3, violet, tan, brown, darkgrey, skyblue, darkorange2, brown4, orangered4, salmon, darkseagreen4, palevioletred3, lightsteelblue1, saddlebrown, honeydew1, skyblue3, darkolivegreen, darkred, thistle1, white in MWCNT_6m; and darkmagenta, steelblue, lightcyan, darkred, orange, darkturquoise, greenyellow, saddlebrown, royalblue, paleturquoise, darkgreen, lightyellow, darkolivegreen, white, pink, darkgrey, darkorange, cyan, grey60, purple, turquoise, gold, brown in MWCNT_12m (Supplementary data 1). Next, the correlations between the gene expression in each module and different doses of 1, 10, 40, 80 µg MWCNT were measured. Afterward, the module-trait analysis to find dose-related modules was performed. Fig S1, S2 and S3 represents the module-trait relationships in which *p*-value < 0.1 determines the modules that remarkably are correlated with individual traits [27]. The module-trait heatmap indicates the correlations of the module eigengenes with traits. The higher correlation shows the increasing eigengene with the increasing trait. In a signed network, it shows that genes of a module follow a similar pattern to increasing expression with increasing trait values. The modules that were significantly correlated to each dose of MWCNT are mentioned in Table 1. This table actually shows that which co-expressed genes as modules are mostly related to the exposure of MWCNT in various doses.

**Table 1:** List of modules that are significantly correlated to each dose of MWCNT. The non-preserved modules which have a remarkable correlation with the trait and highly connected PPI are specified as bold. The parenthesis indicates correlation coefficient and *p*-value

Time	1 month	6 month	12 month
Dose			
1 µg	green (0.31, 0.1)	navajowhite2(-0.46, 0.009), **thistle2** (0.34, 0.06), **tan** (0.31, 0.09), **brown4** (0.3, 0.09), midnightblue (0.3, 0.1)	sienna3 (0.28, 0.1), orange (0.28, 0.1)
10 µg	**white** (0.38, 0.04), **darkturquoise** (0.38, 0.04), steelblue (0.37, 0.05), **mediumpurple3** (0.36, 0.05), saddlebrown (0.35, 0.06), **royalblue** (0.33, 0.08)	**darkslateblue** (0.32, 0.08), darkolivegreen (0.31, 0.09), **lightcyan** (0.3, 0.09)	**royalblue** (0.29, 0.09)
40 µg	orange (0.47, 0.009), **darkolivegreen** (0.45, 0.01), midnightblue (0.45, 0.01), **lightsteelblue1** (0.38, 0.04), turquoise (0.37, 0.05), ivory (0.37, 0.05), magenta (0.35, 0.06), **black** (0.35, 0.06)	**orangered4** (0.33, 0.06), plum1 (0.33, 0.07), yellowgreen (0.31, 0.08)	**darkorange** (0.37, 0.03), **darkgreen** (0.35, 0.04), grey60 (0.33, 0.06), **cyan** (0.32, 0.06), **pink** (0.3, 0.08), **violet** (0.3, 0.08)
80 µg	purple (-0.44, 0.02), red (-0.44, 0.02), **darkorange2** (0.36, 0.05), **darkred** (0.32, 0.09), paleturquoise (0.31, 0.1)	navajowhite2 (0.54, 0.001), **salmon** (0.32, 0.07), purple (0.32, 0.07), grey60 (0.32, 0.08), **lightgreen** 0.31, 0.09), **paleturquoise** (0.31, 0.09), ivory (0.3, 0.1)	**greenyellow** (0.38, 0.02), saddlebrown (0.38, 0.03), purple (0.37, 0.03), **darkred** (0.32, 0.06)

### Protein–protein interactions (PPIs) and pathway enrichment analysis

The PPIs between the genes in each non-preserved module was determined through STRING database. In Table 1, the specific non-preserved and dose-related modules which also had connected PPI are specified as bold and mentioned in Supplementary data 2. These modules contain the genes that were co-expressed after the MWCNT exposure. In the next step, the differentially expressed genes (DEGs) were determined for each dose of MWCNT in different periods using Limma package considering adj.*p*.val < 0.05 and |logFC|> 3 (Supplementary data 3). Afterward, the common genes between DEGs and connected proteins in non-preserved modules were identified (Table 2). The further pathways analysis reveals that the common major genes between co-regulated genes and DEGs dysregulate the pathways related to the metabolism of proteins, immune system, and inflammation (Table 3). From this analysis, we found that although the increase of dose and the period of exposure to MWCNT may lead to dysregulation of more immune and sometimes cancer-related pathways, they affect similar pathways regardless of the MWCNT amount and time of exposure. However, it was found that pathways were disrupted by the function of different differentially expressed and co-expressed genes.

**Table 2:** The common genes between DEGs and connected proteins in the non-preserved modules

Time	1 month	6 month	12 month
Dose			
**1 µg**	–-	**tan (***Krtap19-5*, *Rhoa*, *Rac1***)**, **brown4 (***Ube2l6*, *Actb*, *Prdx2*, *Ctsb*, *Bnip3l***)**	–-
**10 µg**	**white (***Cox8a***)**, **darkturquoise (***Actb*, *Capzb*, *Glrx5*, *Ube2b***)**, **royalblue (***Psme3***)**	**lightcyan (***Gp6*, *Ccni*, *Myh9*, *S100a10***)**	**royalblue (***Ypel5***)**
**40 µg**	**darkolivegreen (***Ezh2***)**, **black (***Fam213a*, *Alas2***)**	**orangered4 (***Ube2k***)**	**darkgreen (***Itgb1***)** , **cyan (***Rap1a***)**, **pink (***Sh3bgrl3*, *Atp5h***)**
**80 µg**	**darkred (***S100a10*, *Ypel5***)**	**salmon (***Ube2b*, *Mbnl1***)**, **lightgreen (***Kbtbd8*, *Riok3*, *Ghitm***)**, **paleturquoise (** *Glrx5***)**	**greenyellow (***Mbnl1***)**

**Table 3:** The enriched pathways by different doses of MWCNT in various periods of time

**1-month exposure**	
**10 µg**	Proteasome Degradation, UCH proteinases, Post-translational protein modification, Deubiquitination, Antigen processing: Ubiquitination & Proteasome degradation, DNA Repair, Class I MHC mediated antigen processing & presentation, Metabolism of proteins, Axon guidance
**40 µg**	Heme Biosynthesis, glycine, serine and threonine metabolic, Metabolism of porphyrins, Lysine degradation, Histone Modifications, PKMTs methylate histone lysines, LncRNA involvement in canonical Wnt signaling and colorectal cancer, Oxidative Stress Induced Senescence, Epigenetic regulation of gene expression, MicroRNAs in cancer
**80 µg**	The TNF-type receptor Fas induces apoptosis on ligand binding., Dissolution of Fibrin Clot, Prostaglandin Synthesis and Regulation, Ciliary landscape, Genes encoding secreted soluble factors
**6-month exposure**	
**1 µg**	Bacterial invasion of epithelial cells, RHO GTPases activate KTN1, EPH-Ephrin signaling, Ras Signaling Pathway, Inflammation mediated by chemokine and cytokine signaling pathway, Focal adhesion, Proteoglycans in cancer, Rap1 signaling pathway, Innate Immune System, mTOR signaling pathway, Pathways Regulating Hippo Signaling, Developmental Biology, TGF-beta Signaling Pathway, Apoptosis, Wnt signaling pathway, Nanomaterial-induced Inflammasome Activation, NOD-like receptor signaling pathway, Chemokine signaling pathway, cAMP signaling pathway, Adaptive Immune System, Signaling by VEGF, intrinsic apoptotic, Transcriptional Regulation by TP53, Nephrin/Neph1 signaling in the kidney podocyte
**10 µg**	The TNF-type receptor Fas induces apoptosis on ligand binding., RHO GTPases activate PAKs, Hemostasis, Prostaglandin Synthesis and Regulation, Nephrotic syndrome, Viral myocarditis, ECM-receptor interaction, Cell surface interactions at the vascular wall, Inflammation mediated by chemokine and cytokine signaling pathway, Fcgamma receptor (FCGR) dependent phagocytosis
**40 µg**	Synthesis of active ubiquitin: roles of E1 and E2 enzymes, Protein ubiquitination, RIG-I/MDA5 mediated induction of IFN-alpha/beta pathways, Antigen processing: Ubiquitination & Proteasome degradation, Class I MHC mediated antigen processing & presentation
**80 µg**	Antigen processing: Ubiquitination & Proteasome degradation, Class I MHC mediated antigen processing & presentation, Synthesis of active ubiquitin: roles of E1 and E2 enzymes, DNA Damage Bypass, Hematopoietic Stem Cell Differentiation, Protein ubiquitination, Adaptive Immune System, Adipogenesis, Ubiquitin mediated proteolysis
**12-month exposure**	
**1 µg**	Cell cycle, Regulation of Apoptosis, p53-Dependent G1 DNA Damage Response, Antigen processing and presentation, MAPK6/MAPK4 signaling, Apoptosis, VEGFR2 mediated cell proliferation, MAPK family signaling cascades, Signaling by Wnt, Metabolism of amino acids and derivatives, Signaling by Interleukins
**10 µg**	Ciliary landscape, Neutrophil degranulation
**40 µg**	VEGFA-VEGFR2 Signaling Pathway, Leukocyte transendothelial migration, Focal adhesion, Rap1 signaling pathway, PTEN dependent cell cycle arrest and apoptosis, adenosine ribonucleotides de novo biosynthesis, Signaling by Interleukins, MAP2K and MAPK activation, IFN-gamma pathway, Hemostasis, Class I PI3K signaling events, Cytokine Signaling in Immune system, Oncogenic MAPK signaling, Electron Transport Chain, Developmental Biology, Oxidative phosphorylation, TGF-beta Signaling Pathway, ECM-receptor interaction, glomerulonephritis, Genes controlling nephrogenesis
**80 µg**	Adipogenesis

## Discussion

One of the major challenging in the recent decade is the adverse effects of nanomaterials like MWCNT on the environment and human health. The increasing application of MWCNT in the industry makes the concern about the detrimental consequences of exposure. MWCNT has similar pathogenicity effects to asbestos fibers such as carcinogenic and profibrotic risk due to their resembling structures [28]. However, there is rare information about the dysregulated proteins and pathways due to MWCNT.

In this study, we explored toxicity pathways which are defined as the cellular response pathways that lead to a detrimental health effect when adequately perturbed. Systems toxicology provides a useful approach to determine the association between toxicity and the changed expression of a set of genes. One of the procedures to investigate the systems toxicology is finding co-expressed genes which contribute to a biological process. Moreover, a toxicity pathway can be determined through the identification of a set of co-expressed genes that are activated in response to the MWCNT exposure [29]. To determine these functional pathways, the weighted gene co-expression network and differentially expressed gene analyses were utilized.

The pathway enrichment analysis disclosed that different doses of MWCNT cause the activation of inflammation-, immune-, and carcinogenic-related pathways including Antigen processing: Ubiquitination & Proteasome degradation, Class I MHC mediated antigen processing & presentation, chemokine and cytokine signaling pathway, TGF-beta Signaling Pathway, MAPK family signaling cascades, apoptosis, immune system, oxidative stress, Transcriptional Regulation by TP53, VEGFA-VEGFR2 Signaling Pathway as well as metabolic pathways. In the following, we discuss the involvement of various proteins in the mentioned activated pathways due to MWCNT exposure.

There are several studies that reported the cytotoxicity effect of carbon nanotubes with disruption of the immune system [30–33]. The immune system is extensively affected by the MWCNT, which is concluded by the activation of Adaptive Immune System (UBE2B, KBTBD8), Innate Immune System (RHOA, CTSB, RAC1, ACTB, UBE2L6), and Cytokine Signaling in Immune system (RAP1A and ITGB1). Antigen processing: Ubiquitination & Proteasome degradation and Class I MHC mediated antigen processing & presentation are the pathways that were mainly activated by deregulation of ACTB, PSME3, UBE2B, UBE2K, and KBTBD8. It has been found that the designed nanomaterial may promote antigen processing and presentation utilizing a dual-trafficking route: cross-presentation and exogenous pathway [34]. Moreover, the proteasome system has a substantial efficacy in immune regulation through different mechanisms containing MHC class I antigen processing and regulation of inflammation/cytokine production [35].

It also has been disclosed that MWCNT and SWCNT induce the secretion of inflammatory factors, chemokines, and growth factors such as transforming growth factor (TGF)-b1 and tumor necrosis factor (TNF)-a in mouse macrophages [33, 36–38]. In this study, various inflammation pathways were also enriched with the involvement of several hub genes related to different doses of MWCNT including Inflammation mediated by chemokine and cytokine signaling pathway (RHOA, RAC1, ACTB, MYH9), TGF-beta Signaling Pathway (RHOA, RAC1, ITGB1), MAPK family signaling cascades (PSME3 and RAP1A), Oncogenic MAPK signaling (RAP1A), Transcriptional Regulation by TP53 (PRDX2 and BNIP3L), and Neutrophil degranulation (YPEL5). MWCNT induces inflammation and fibrosis through the liberalization of inflammatory cytokines from macrophages [39]. The MAPK pathway is a significant signal transduction pathway that governs a series of events that persuade gene expression relevant to inflammation, apoptosis, and fibrosis. This pathway may also be induced by carbon nanotube exposure [40]. MWCNT can also activate the TGF-β signaling pathway and induce the TGF- 1 production in macrophages, fibroblasts, and epithelial cells [41].

The carcinogenesis and genotoxic effects of CNTs such as oxidation of DNA base, breaking DNA strand, and also clastogenic and aneugenic effects have also been reported [42]. Herein, the dysregulation of pathways including DNA repair (PSME3 and ACTB), DNA Damage Bypass (UBE2B), and p53-Dependent G1 DNA Damage Response (PSME3) by the effect MWCNT were also observed. DNA damage induced by MWCNTs can be developed through downregulation of related genes which helps to the development of carcinogenicity [43]. Another enriched cancer-related pathway is VEGFA-VEGFR2 Signaling Pathway which was enriched by RAP1A, SH3BGRL3, and ITGB1. It is the main pathway that activates angiogenesis by inducing the survival, proliferation, and migration of endothelial cells. The recent studies also showed the modulation of CNT on the proliferation of various types of cells in animals [44].

Previous studies demonstrated that apoptosis could be induced in mitochondria by MWCNTs, possibly through two major mechanisms, including oxidative stress and mitochondrial membrane potential. Oxidative stress is implicated through the liberation of pro-inflammatory mediators [45]. The oxidative stress toxicity may also induce apoptosis by SWCNTs, leading to activating signals of p53-mediated DNA damage checkpoint and then apoptosis. Apoptosis is one of the enriched pathways by different doses of MWCNT through various pathway mechanisms including TNF-type receptor Fas induces apoptosis on ligand binding (S100A10), intrinsic apoptotic (BNIP3L), Regulation of Apoptosis (PSME3), PTEN dependent cell cycle arrest and apoptosis (ITGB1), and Apoptosis (CTSB and ACTB) [46, 47]. Oxidative stress may also be induced by the disturbed amino acids and the involved pathways. It has been found that nano-TiO_2_ can disrupt the metabolism of amino acids, inhibit the RNA and DNA synthesis, and damage energy production [48]. Similarly, the dysregulation of metabolism of proteins (ALAS2, UBE2B, ACTB), as well as various pathways related to degradation and modification of amino acids such as glycine, serine and threonine metabolic (ALAS2), Lysine degradation (EZH2), Metabolism of amino acids and derivatives (PSME3), and also adenosine ribonucleotides de novo biosynthesis (ATP5H) were observed. Moreover, the synthesis and ubiquitination of proteins are largely affected by changes in the expression levels of UBE2B, UBE2K, KBTBD8, PSME3, and ACTB [49, 50]. CNTs in the body can increase the level of free radicals leading to oxidative stress and oxidation of DNA, proteins, and lipids [51]. ROSs increase the oxidation of amino acids, inactivation of enzymes, and apoptosis [7, 51].

The nephrotoxicity of nanoparticles has also been reported. Some NPs may cause mitochondria and cell membrane perturbation as well as disturbance of the energy metabolism in rat kidneys [52]. Likewise, the dysregulation of pathways including glomerulonephritis and Genes controlling nephrogenesis (ITGB1), Nephrin/Neph1 signaling in the kidney podocyte (RAC1), and Nephrotic syndrome (MYH9) were also determined in this study due to the cytotoxicity effect of MWCNT.

Adipogenesis is the process by which adipocytes develop and accumulate as adipose tissue at different sites in the body. There are several studies that reported graphene and graphene oxide inhibited and enhanced adipogenesis, respectively [53]. Indeed, graphene oxide has a strong affinity towards insulin, which ultimately induces adipogenesis. We also found the dysregulation of adipogenesis pathway by deregulation of Mbnl1.

Moreover, the modulatory effect of carbon nanomaterials on stem cell differentiation has been reported [54]. The activation of Hematopoietic Stem Cell Differentiation pathway was also observed by deregulation of RIOK3. Hemostasis was also activated by the change in the expression levels of GP6, S100A10, RAP1A, and ITGB1 due to the MWCNT exposure. It is in agreement with the previous reports regarding the effect of nanomaterials in developing hemostasis [55, 56].

Generally, we did not find a comprehensive association between various doses of MWCNTs and the resulted dysregulated pathways. However, disparate genes participate in the dysregulation and activation of pathways. Our study has some limitations. The co-expressed gene groups were recognized by analysis of the mouse microarray dataset. Further in vitro studies must be carried out to validate the identified genes associated with MWCNT. Moreover, the analysis of more samples improves the reliability of WGCNA analysis and possibly finding the dose-related genes. The outcomes of WGCNA may technically be biased because of tissue contamination. However, this work partially clarifies the gene groups affected by MWCNT that should be considered for study the affected biological pathways.

## Conclusion

The outcomes of this study reveal that regardless of the exposure dose, MWCNTs can induce genotoxic and carcinogenic effects. Although our study has some limitations it introduces several novel dysregulated genes that have important roles in the dysregulation of carcinogenic- and cytotoxic- related pathways. These genes should be further validated in the human cells in a large sample size.

## Methods

### Dataset and preprocessing

**Fig. 2:**
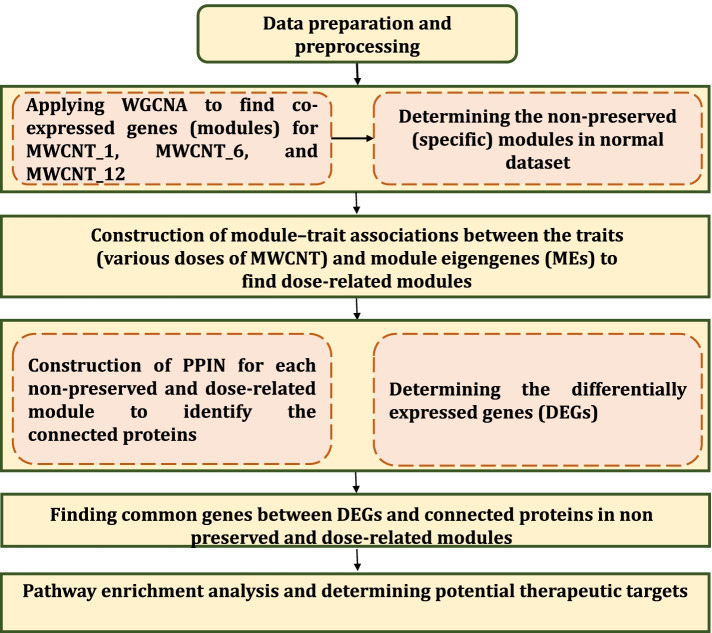
The flowchart containing the suggested procedure utilized in this study

A gene expression microarray dataset with accession number GSE126959 [57] was downloaded from NCBI Gene Expression Omnibus (GEO) database. It contains the microarray gene expression profiles in mouse blood after 1-month (29 samples), 6-month (32 samples), and 12-month (35 samples) aspiration exposure to different doses (1, 10, 40, and 80 µg) of MWCNT. The gene expression profile of dispersion media (DM) control was also measured after one month (7 samples), six months (8 samples), and twelve months (9 samples). The MWCNT doses in mice were chosen to approximately be equivalent to the human occupational exposures [57]. For example, 10 µg MWCNT exposure in mice is almost equal to the deposition for a person doing work for about one month in a workplace with MWCNT aerosol of 400 µg/m^3^ or 9 months to 7.5 years with 4–40 µg/m^3^. Therefore, the employed MWCNT doses for mice approximately simulate the human occupational exposures to MWCNT. A total of 24 525 genes were firstly considered for analysis. The dataset was quantile normalized and log2 transformed. The flowchart containing the suggested procedure is depicted in Fig. 2.

### Construction of weighted co-expression network and identification of modules

In order to construct the weighted co-expression networks, the WGCNA package in the R environment was employed [18]. Firstly, the “goodSamplesGenes” function was used to remove genes with exceeding missing values. After preprocessing with goodSamplesGenes function and removing unqualified genes, 24 378, 24 466, and 24 497 genes were considered in MWCNT_1m, MWCNT_6m, and MWCNT_12m groups for further analyses. Next, the outlier samples were explored using “hclust” function. Next, the “pickSoftThreshold” function was applied to find scale-free topology fitting indices (R^2^) versus various soft thresholding powers *β*. The threshold of R^2^ ≥ 0.8 was considered to choose the *β* value. Afterward, the pairwise correlation between genes was computed and then transformed into an adjacency matrix. Topological Overlap Matrix (TOM) and dissimilarity of TOM (1-TOM) were then determined. After that, the “hclust” function was employed to generate a hierarchical clustering tree (dendrogram) of genes based on dissTOM. The “cutreeDynamic” function was then used to cut the branches. Next, module eigengene (ME) that is the first principal component of a given module, was determined. The “mergeCloseModules” function was then utilized to merge close modules considering the height threshold of 0.25 [58].

### Identification of non-preserved modules

Through module preservation analysis, the modules that were non-preserved in the DM control were determined. To this end, the “modulePreservation” function in WGCNA package and permutation-based statistics to determine Z_summary_ and medianRank scores were used. The Z_summary_ measures both aspects of density and connectivity preservation [59, 60]. Generally, a module with lower Z_summary_ and higher medianRank has a low tendency to be preserved. Here, a module with Z_summary_ < 2 and medianRank ≥ 8 was interpreted as non-preserved, a module with 2 < Z_summary_ ≤ 8 and medianRank < 8 was considered as semi-preserved, and a module with Z_summary_ > 10 and medianRank < 8 was defined as highly preserved [61]. For the modulePreservation function, some parameters (nPermutations = 200, maxModuleSize = 100) were set, whereas others were left as default.

### Construction of module–trait association

The correlation between the traits (various doses of MWCNT) and module eigengenes (MEs) was determined as the module–trait association. To this end, “cor “ and “corPvalueStudent” functions in WGCNA package were employed. The *p*-value < 0.1 was considered to find meaningful associations.

### Protein–protein interactions (PPIs) and enrichment analysis

The biological associations between proteins were identified utilizing the STRING database [62]. To this purpose, the proteins were submitted in STRING and the interactions.

with a combined score > 0.4 were considered as the cut-off criterion. In order to find the biological pathways enriched by hub genes in each module, ToppGene webtool was utilized [63]. To this end, ToppFun tools was used. It discovers the functional enrichment of input genes according to Transcriptome. Top pathway terms with a *p*-value < 0.05 were considered for further interpretations.

## Supplementary Information

**Additional file 1. **

**Additional file 2. **

**Additional file 3. **

**Additional file 4. **

**Additional file 5. **

**Additional file 6. **

## Data Availability

The datasets used and/or analyzed during the current study are available at GEO database: https://www.ncbi.nlm.nih.gov/geo/query/acc.cgi?acc = GSE126959. Programming language: R. Other requirements: R environment. R. Packages: GEOquery, Limma. Tested on R version 3.6.1.
