# Supplementary figures and images for: Evaluating the cytotoxicity and pathogenicity of multi-walled carbon nanotube through weighted gene co-expression network analysis: a nanotoxicogenomics study
Source: BMC Genom Data. 2022 Feb 17;23:12. doi: 10.1186/s12863-022-01031-3 (PMC8851761; doi:10.1186/s12863-022-01031-3)

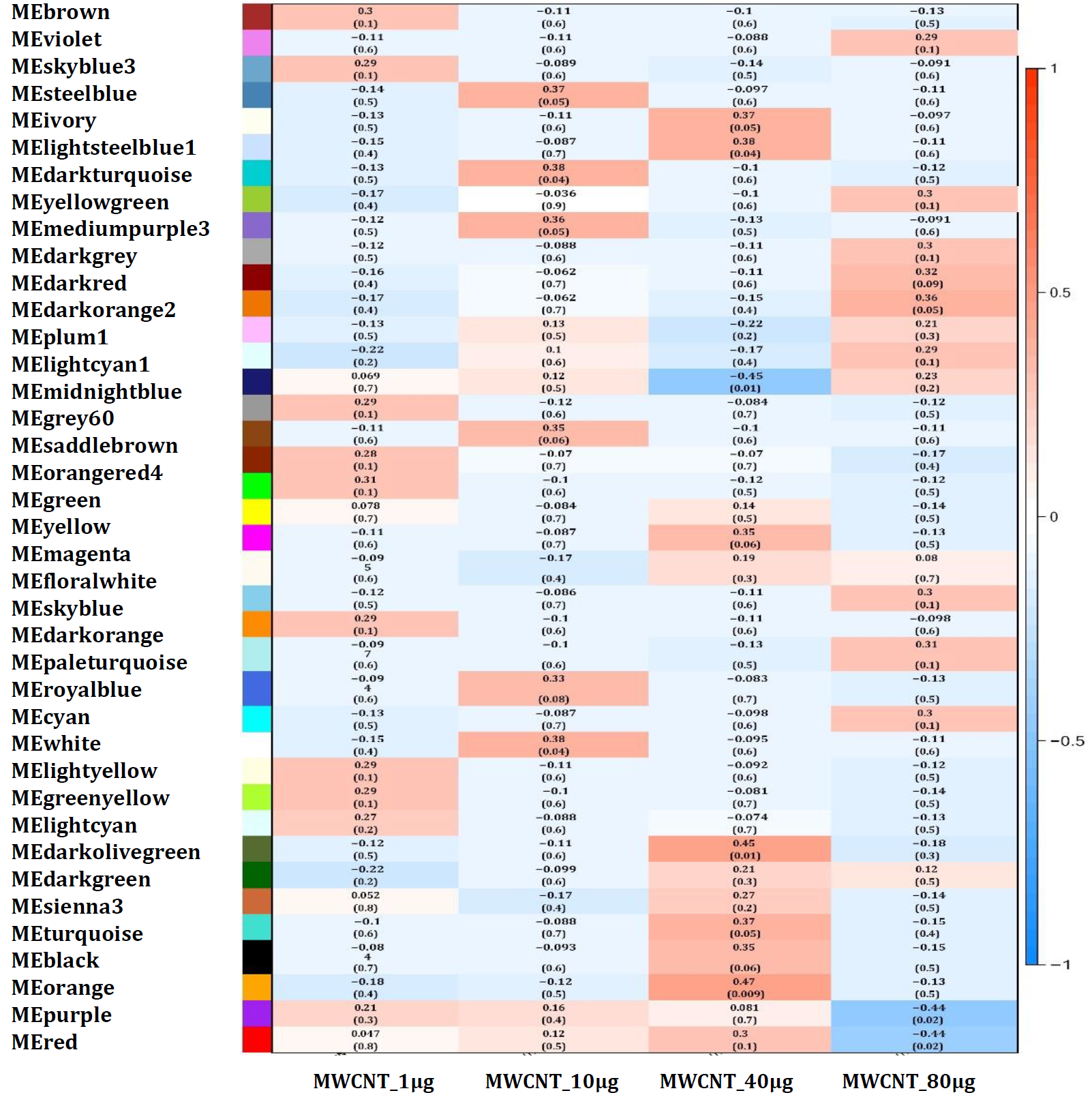

Supplement: Supplementary file 2 — Additional file 2. [file 12863_2022_1031_MOESM2_ESM.tif]

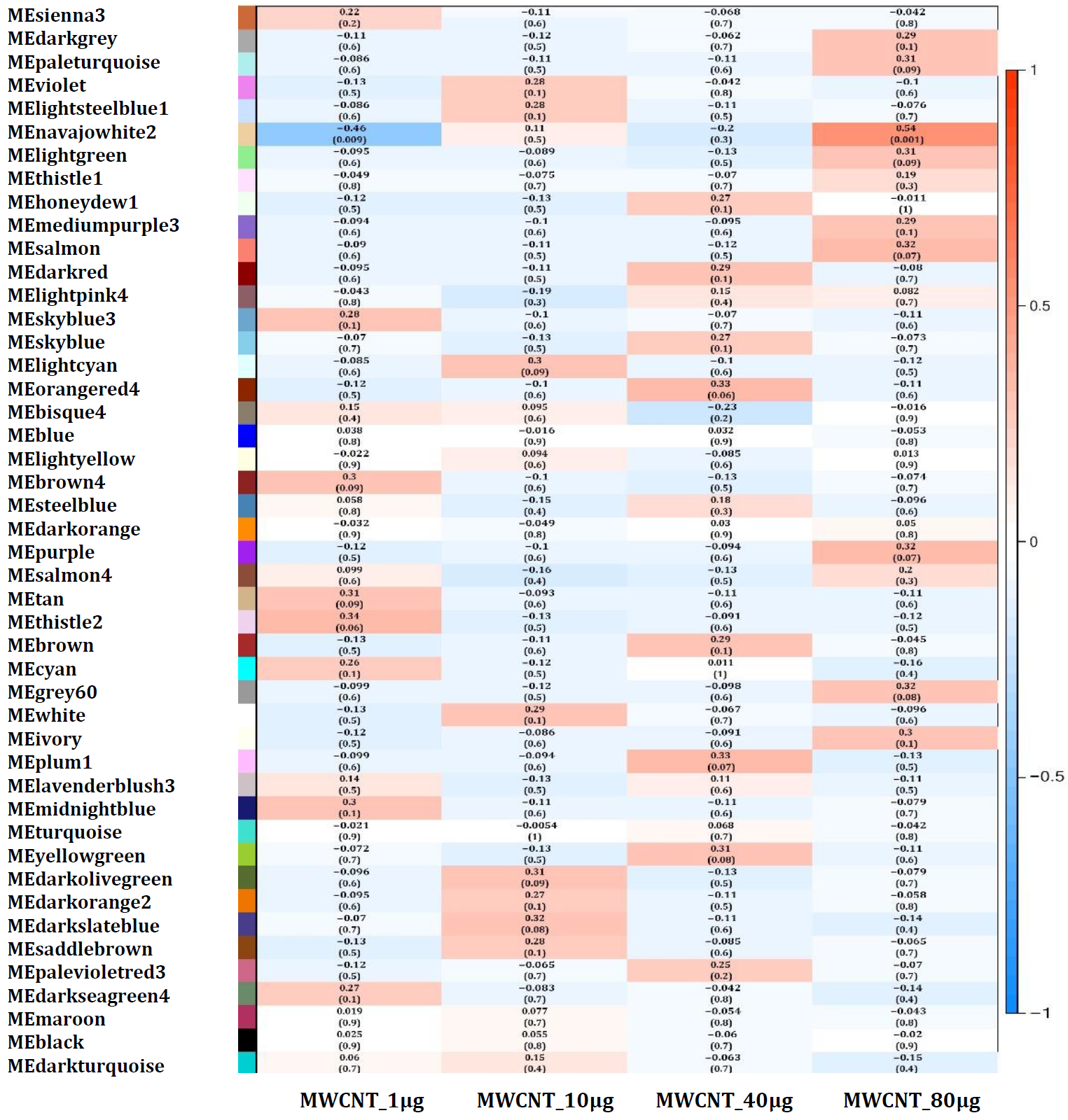

Supplement: Supplementary file 3 — Additional file 3. [file 12863_2022_1031_MOESM3_ESM.tif]

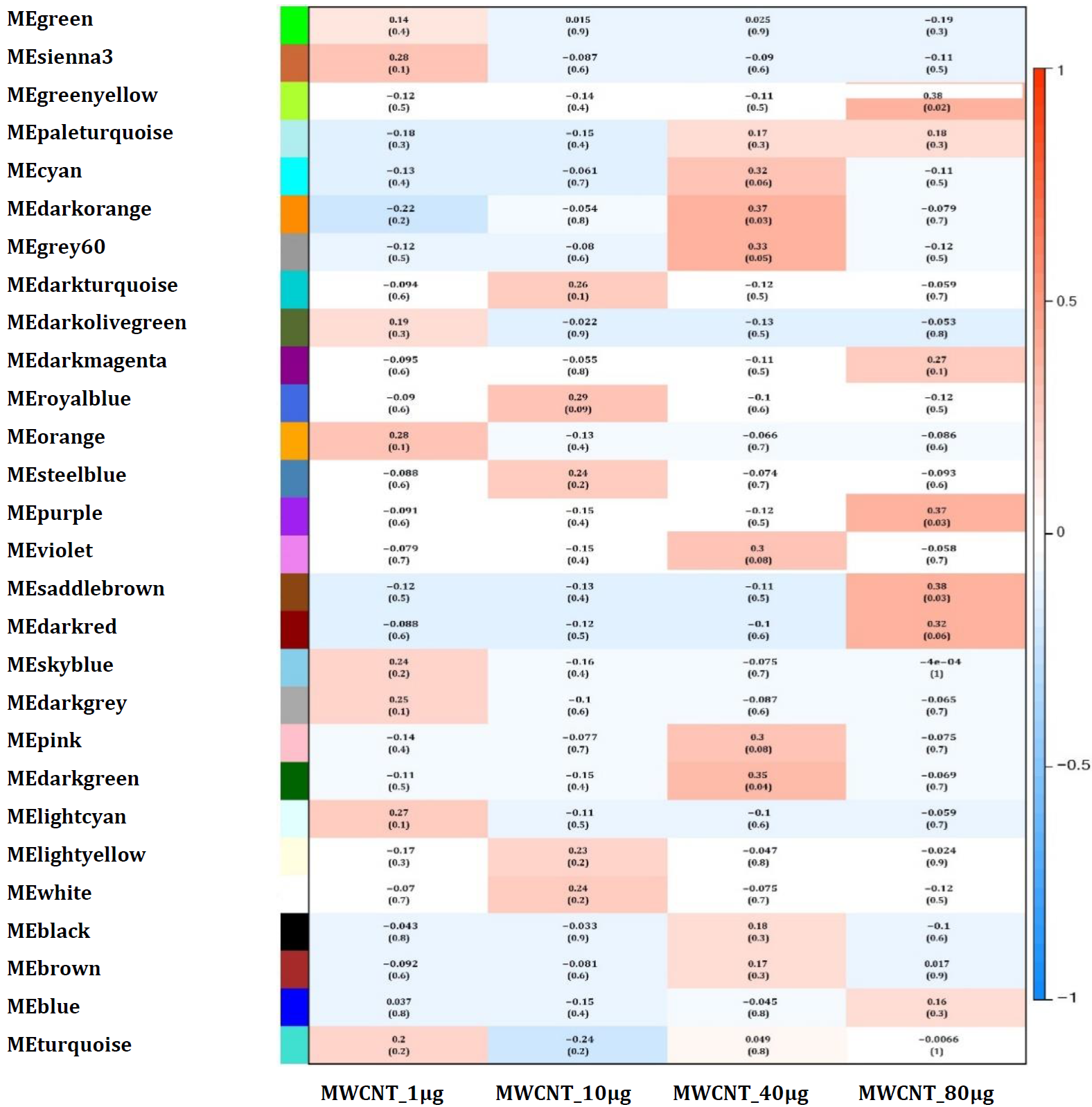

Supplement: Supplementary file 4 — Additional file 4. [file 12863_2022_1031_MOESM4_ESM.tif]
